# Fabrication and Characteristics of Sintered Cutting Stainless Steel Fiber Felt with Internal Channels and an Al_2_O_3_ Coating

**DOI:** 10.3390/ma11030455

**Published:** 2018-03-20

**Authors:** Shufeng Huang, Zhenping Wan, Shuiping Zou

**Affiliations:** 1 School of Mechanical and Automotive Engineering, South China University of Technology, Guangzhou 510640, China; sfhuang56@126.com (S.H.); meshpzou@mail.scut.edu.cn (S.Z.); 2 Department of Mechanical Engineering, University of South Carolina, Columbia, SC 2910, USA

**Keywords:** porous materials, sintered cutting stainless-steel fiber felt, adhesive strength, permeability

## Abstract

A novel sintered cutting stainless steel fiber felt with internal channels (SCSSFFC) composed of a stainless-steel fiber skeleton, three-dimensional interconnected porous structure and multiple circular microchannels is developed. SCSSFFC has a jagged and rough surface morphology and possesses a high specific surface area, which is approximately 2.4 times larger than that of the sintered bundle-drawing stainless steel fiber felt with internal channels (SBDSSFFC) and is expected to enhance adhesive strength. The sol-gel and wet impregnation methods are adopted to prepare SCSSFFC with an Al_2_O_3_ coating (SCSSFFC/Al_2_O_3_). The adhesive strength of SCSSFFC/Al_2_O_3_ is investigated using ultrasonic vibration and thermal shock tests. The experimental results indicate that the weight loss rate of the Al_2_O_3_ coating has a 4.2% and 8.42% reduction compared with those of SBDSSFFCs based on ultrasonic vibration and thermal shock tests. In addition, the permeability of SCSSFFC/Al_2_O_3_ is investigated based on forced liquid flow tests. The experimental results show that the permeability and inertial coefficients of SCSSFFC/Al_2_O_3_ are mainly affected by the coating rate, porosity and open ratio; however, the internal microchannel diameter has little influence. It is also found that SCSSFFC/Al_2_O_3_ yields superior permeability, as well as inertial coefficients compared with those of other porous materials reported in the literature.

## 1. Introduction

Volatile organic compounds (VOCs) emitted from petrochemical and chemical industries, printing presses and transport vehicles have been a major source of air pollution [1,2]. Catalytic combustion is one of the most effective methods for the elimination of VOCs because of its high purification efficiency and low energy consumption [3,4]. Structured catalysts for catalytic combustion usually consist of a monolithic porous support, porous coating and active components. The porous support plays an important role that influences the flow distribution, heat transfer, pressure drop, catalytic performance and service life [5,6,7]. 

Monolithic porous supports are typically made of ceramics or metals [8,9]. Currently, substantial attention has been paid to metallic supports, especially those made of stainless steel, because of their good corrosion resistance, privileged mechanical strength, high thermal conductivity and low manufacturing cost [10,11]. In addition to metallic honeycombs [12,13], many other kinds of metallic supports, such as knitted wire gauze [14] and stainless steel wire cloth [15], have been proposed to achieve a high inertial coefficient as well as good permeability, good heat and mass transfer. However, there is one underlying problem concerning the use of metal substrates as supports—their slick surface and small specific surface area, which results in poor adhesion and non-uniformity of coatings [15]. To achieve a rough surface and a large surface area, Korkmaz [16] investigated the open cell Ti6Al4V alloy foams with an Al_2_O_3_ coating based on micro-arc oxidation and it is found out that the mechanical properties of the foam materials were significantly improved. Tronconi et al. [17] addressed several aspects of catalytic coating deposition on metallic foams with a complex reticulated structure. Many other attempts have been tried, such as the wet oxidation process [18,19], chemical vapor deposition [20], anodic oxidation [21,22], thermal oxidation [2] and wet etching [23]. However, these treatment methods are time consuming and not cost effective, which restricts their practical applications. Thus, it is of urgency to develop an effective and promising preparation technology to increase the adhesive strength of Al_2_O_3_ coatings by improving the superficial roughness of metallic supports.

In this work, a sintered cutting stainless steel fiber felt with internal channels (SCSSFFC) is developed based on the bifurcating chip cutting method. The cutting stainless steel fiber is prepared by the bifurcating chip cutting method. The manufacturing processes of sintered cutting stainless-steel fiber felt with internal channels and an Al_2_O_3_ coating (SCSSFFC/Al_2_O_3_) are presented and the characteristics and surface morphology of SCSSFFC/Al_2_O_3_ made with different types of fibers are analyzed. The effect of the surface morphology on adhesive strength of SCSSFFC/Al_2_O_3_ is studied using ultrasonic vibration and thermal shock tests. Moreover, the influences of the Al_2_O_3_ coating rate and the structural parameters of the SCSSFFC on the permeability and inertial coefficient of SCSSFFC/Al_2_O_3_ are investigated experimentally and the modified correlations are put forward to predict the permeability and inertial coefficient of SCSSFFC/Al_2_O_3_.

## 2. Experimental Procedures

### 2.1. Fabrication of the SCSSFFC with Al_2_O_3_ Coating

**Figure 1:**
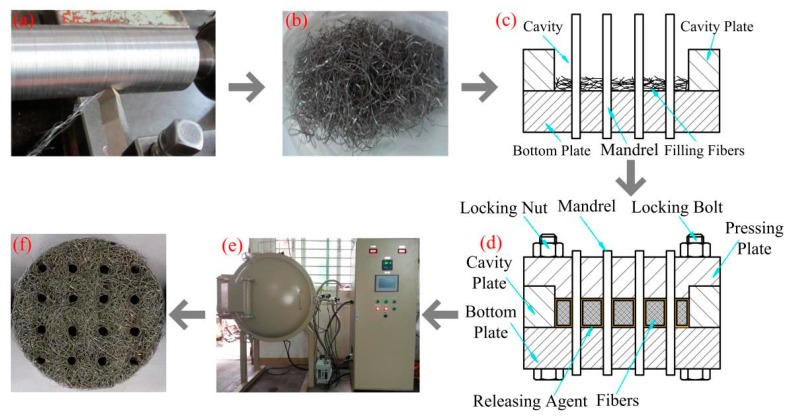
Manufacturing processes of sintered cutting stainless steel fiber felt with internal channels (SCSSFFC): (**a**) cutting; (**b**) clipping; (**c**) filling; (**d**) pressing and preforming; (**e**) sintering and (**f**) sample.

The preparation of SCSSFFC includes the following steps: long and rough stainless steel fiber manufacturing, fiber clipping, short stainless steel fiber bedding, mold pressing and sintering as shown in Figure 1. Based on the bifurcating chip cutting method previously reported [24], the long stainless steel fibers with rough surface morphologies are fabricated on the precise lathe C6132A, presented in Figure 1a. Subsequently, the long stainless steel fibers with an equivalent diameter of 130 μm are segmented into short fibers of 10–20 mm in length, as shown in Figure 1b. Following, the short fibers are bedded into a cavity formed between mandrels and cavity plate layer by layer disorderly and uniformly as shown in Figure 1c. The sintering mold consists of molybdenum mandrels, a bottom plate with mandrel holes, a cavity plate and a pressing plate with mandrel holes. The bottom plate, cavity plate and pressing plate are made of high-temperature nickel-based alloys. Then, the pressing plate is tightened with locking nuts, as shown in Figure 1d. Afterwards, the semi-finished sample is sintered in a vacuum furnace at a temperature of 1200 °C for 60 min, as shown in Figure 1e. When the furnace is cooled down to 200 °C, the SCSSFFC samples are taken out and cooled to room temperature, as shown in Figure 1f. Hereafter, the sintered cutting stainless steel fiber felt with internal channels is named as SCSSFFCs. The sintered bundle-drawing stainless steel fiber felt with internal channels is named as SBDSSFFCs.

The average porosity of an SCSSFFC can be calculated by the quality-volume method [25], as shown in the following equation:

(1) $$\varepsilon =1-4\frac{m}{\rho^{\prime }\pi D^{2}H}$$

where *m*, *D* and *H* are the mass, diameter and thickness of the SSFSFHC sample, respectively; and *ρ*’ is the density of 1Cr18Ni9Ti. In the present study, the thickness and diameter of the SCSSFFCs are 8 mm and 50 mm, respectively.

The open ratio of samples is defined as the ratio of the total cross-sectional area of the microchannels to the open region area in an SCSSFFC, which can be expressed by:

(2) $$\alpha =\frac{Nd^{2}}{D^{2}}$$

where *N* and *d* are the number and diameter of the microchannels, respectively.

Sol-gel and wet impregnation methods [26,27] are adopted to coat Al_2_O_3_ onto the SCSSFFCs. An SCSSFFC is dipped into the Al_2_O_3_ sol prepared by the hydrolysis of aluminum isopropoxide in advance and then pulled from the sol and dried. After the drying process, the pretreated SCSSFFC is placed in a muffle furnace and calcined at 500 °C for 120 min. The above steps are repeated until the Al_2_O_3_ coating reaches a specified amount. Then, the SCSSFFC with an Al_2_O_3_ coating can be obtained. Hereafter, an SCSSFFC with an Al_2_O_3_ coating will be called SCSSFFC/Al_2_O_3_. An SDBSSFFC with an Al_2_O_3_ coating will be called SBDSSFFC/Al_2_O_3_. The coating rate of a sample is defined as follows. Whether SCSSFFC or SDBSSFFC, the coating rate of the samples is calculated the same.

*β* = (*w*_2_ − *w*_1_)/*w*_1_ (3)

where *w*_1_ is the initial weight of a sample before coating, *w*_2_ represents the weight of a sample after finally coating and *β* is the coating rate of samples. 

### 2.2. Adhesive Strength Test for SCSSFFC/Al_2_O_3_

To evaluate the effect of surface morphology on the adhesive strength of SCSSFFC/Al_2_O_3_, ultrasonic vibration and thermal shock tests [28,29] are conducted. In a typical ultrasonic vibration testing, the SCSSFFC/Al_2_O_3_ samples are immersed in a sealed beaker full of ethanol and then treated in an ultrasonic bath (SK2200H, Shanghai, China) for 90 min, followed by drying in a furnace at 120 °C for 120 min. Finally, the weight loss is measured. The thermal shock tests are carried out by heating the SCSSFFC/Al_2_O_3_ samples to 800 °C for 20 min and then immediately quenching them in water at 25 °C. The thermal shock test is repeated 10 times for each sample. In ultrasonic vibration and thermal shock tests, the weight loss rate of a sample is used to evaluate the adhesive strength of the Al_2_O_3_ coating to SCSSFFC which is defined by

*φ*= (*m*_1_ − *m*_2_)/*m*_1_ (4)

where *m*_1_ is the initial weight of SCSSFFC/Al_2_O_3_ before the test, *m*_2_ represents the weight of SCSSFFC/Al_2_O_3_ after the test and *φ* is the weight loss rate. 

### 2.3. Permeability Test for SCSSFFC/Al_2_O_3_

**Figure 2:**
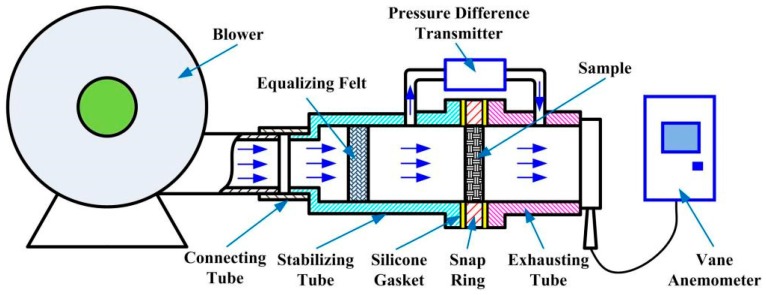
Schematic of the experimental apparatus for measuring the pressure drop across SCSSFFC/Al_2_O_3_.

As shown in Figure 2, the experimental setup for the permeability of SCSSFFC/Al_2_O_3_ is composed of a blower, connecting tube, stabilizing tube, snap ring, exhausting tube, pressure difference transmitter and a vane anemometer. The SCSSFFC/Al_2_O_3_ sample is embedded in the snap ring placed between the stabilizing tube and exhausting tube. To avoid air flow leakage, two pieces of silicone gasket are covered on the two end faces of the snap ring. In a typical experiment, the first step is to test the air tightness of the setup. Then, the air flow is blown into the stabilizing tube by the blower and flown into the equalizing felt to develop the air flow sufficiently and uniformly. Afterwards, the air flow passes through the tested samples and discharges into the atmosphere through the exhausting tube. The air velocity is set to a desired value by adjusting the blower. When the test system reaches steady state, the pressure drop across the SCSSFFC/Al_2_O_3_ sample is measured by the pressure difference transmitter. The average air velocity at the outlet of the exhausting tube is recorded by the vane anemometer. 

The quadratic Forchheimer equation [30], shown as follows, is adopted to address the experimental results.

(5) $$\frac{\Delta P}{H}=\frac{\mu }{K}u+F_{K}\rho u^{2}$$

where *K* and *F*_K_ are the permeability and the inertial coefficients, respectively; *H* is the thickness of the porous material; *µ* the dynamic viscosity of air; *u* the air velocity; *P* and *ρ* are the pressure and density of air, respectively.

To conveniently obtain the permeability and inertial coefficient of the SCSSFFC/Al_2_O_3_, Equation (4) can be transformed into

(6) y=a⋅u+b

where the variables *y*, *a* and *b* are defined as follows:

(7) $$y=-\frac{\Delta P}{H}\cdot \frac{1}{u}$$

(8) $$a=F_{K}\cdot \rho$$

(9) $$b=\frac{\mu }{K}$$

where Δ*P* and *H* are the pressure drop across the sample and the thickness of the sample, respectively. Then, a least square fitting of a linear function of *u* is used to determine the values of *a* and *b*. Therefore, *K* and *F*_K_ are determined by the slope and *y*-axis intercept of the linear function.

### 2.4. Uncertainty Analysis

The whole uncertainty includes the measurement errors of the pressure drop (δΔ*P*), air flow velocity (δ*u*) and sample thickness (δ*H*). The uncertainties of the pressure drop, air flow velocity and sample thickness are decided by the measurement accuracy of the pressure difference transmitter, vane anemometer and the vernier caliper, respectively. Hence, they can be estimated as

(10) $$\frac{\delta \Delta P}{\Delta P}=0.002$$

(11) $$\frac{\delta u}{u}=0.05$$

(12) $$\frac{\delta H}{H}=0.0025$$

Then, the uncertainties in the values of *K* and *F*_K_ can be calculated as

(13) $$\frac{\delta K}{K}=\sqrt{{\left(\frac{\delta \Delta P}{\Delta P}\right)}^{2}+{\left(\frac{\delta u}{u}\right)}^{2}+{\left(\frac{\delta H}{H}\right)}^{2}}=5.01\%$$

(14) $$\frac{\delta F_{K}}{F_{K}}=\sqrt{{\left(\frac{\delta K}{K}\right)}^{2}+{\left(\frac{\delta \Delta P}{\Delta P}\right)}^{2}+2{\left(\frac{\delta u}{u}\right)}^{2}+{\left(\frac{\delta H}{H}\right)}^{2}}=8.67\%$$

## 3. Results 

### 3.1. Characteristics of SCSSFFC/Al_2_O_3_

**Figure 3:**
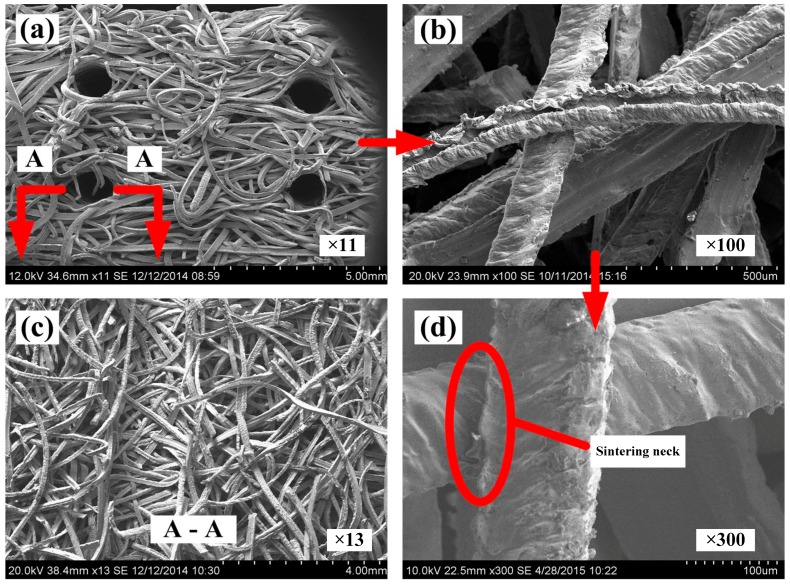
Scanning electron microscopy (SEM) of SCSSFFC: (**a**) appearance; (**b**) rough topography; (**c**) the inner wall structure of the honeycomb channel; (**d**) sintering neck.

**Figure 4:**
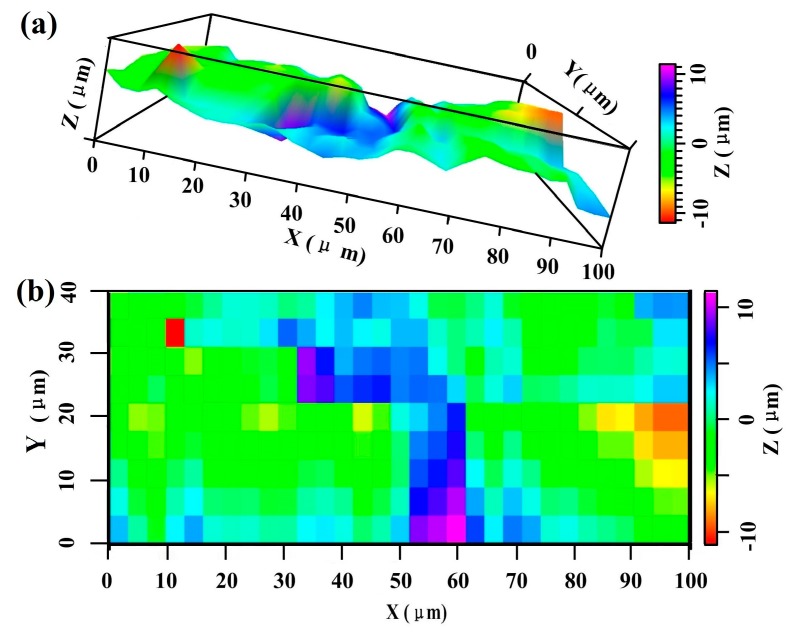
Surface morphology of single cutting fiber: (**a**) surface profile; (**b**) surface contour nephogram.

**Figure 5:**
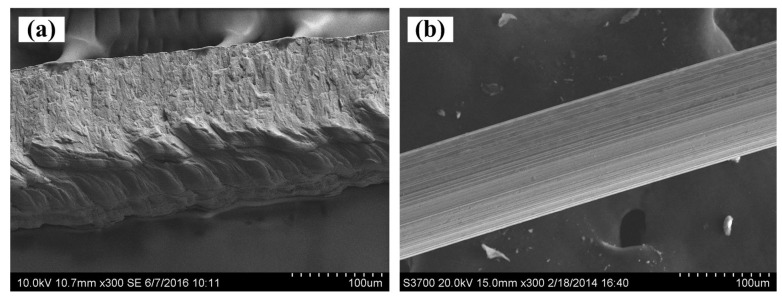
SEM of single cutting stainless steel fiber (**a**) and bundle-drawing fiber (**b**).

The scanning electron microscope (SEM, Hitachi S-3700, Tokyo, Japan) images of SCSSFFC are shown in Figure 3. SCSSFFC is composed of stainless steel fibers skeleton, three-dimensionally interconnected porous structure and multiple circular microchannels, as presented in Figure 3a. The inner wall of the interconnected microchannels has a three-dimensional porous structure with bulges and sags (Figure 3c), which is helpful to increase radial mixing and increase the permeability compared with other monolithic supports. On the other hand, it can be seen from Figure 3b,d that the cutting stainless steel fiber has a rough surface morphology induced by shear deformation during the manufacturing processes. Figure 4 presents the surface morphology of the single cutting measured by a 3D surface morphology analyzer (BMT EXPERT, Tuttlingen, Germany). The relief height of the surface microstructure is up to 22 μm. Analysis of the SEM images in Figure 5 reveals that the single cutting stainless steel fiber has rough surface compared with the bundle-drawing fiber. The nitrogen adsorption method [31] is used to measure the specific surface area of SCSSFFCs. The specific surface area of the smooth SBDSSFFC is 0.0786 m^2^/g, while the specific surface area of the rough SCSSFFC is 0.2654 m^2^/g, which is approximately 2.4 times larger than that of the SBDSSFFC made by bundle-drawing fiber. Therefore, this indicates that the higher specific surface area is beneficial to increase adhesive strength of the coating to the support.

**Figure 6:**
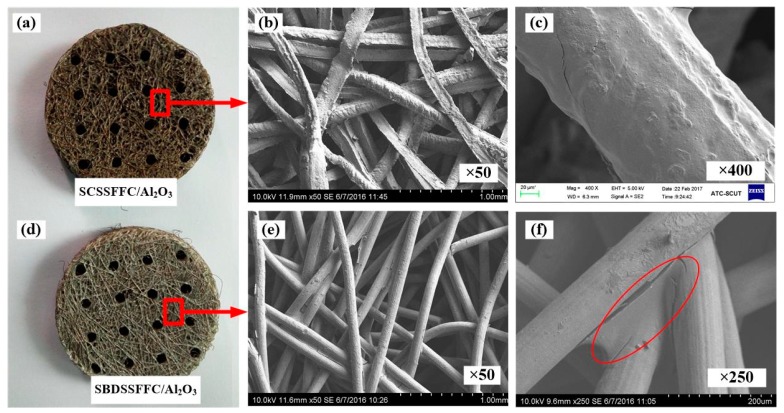
Different samples with Al_2_O_3_ coating: (**a**) appearance of SCSSFFC/Al_2_O_3_ (**b**,**c**) SEM of SCSSFFC/Al_2_O_3_; (**d**) appearance of SBDSSFFC/Al_2_O_3_; (**e**–**f**) SEM of SBDSSFFC/Al_2_O_3_.

The appearance and morphology of the different samples with the Al_2_O_3_ coating are shown in Figure 6. For comparison, SBDSSFFC/Al_2_O_3_ made of commercial bundle-drawing stainless steel fibers with a diameter of 100 µm is also prepared by the same preparation process. It can be found that the Al_2_O_3_ is coated on the surface of the cutting stainless steel fibers and the pores are not clogged. After coating Al_2_O_3_, the rough surface morphologies of SCSSFFC/Al_2_O_3_ are still evident as shown in Figure 6b,c. For the smooth SBDSSFFC/Al_2_O_3_, the Al_2_O_3_ coating is apt to accumulate adjacent to the sintering joints as shown in Figure 6e,f. Therefore, the rough SCSSFFC can improve the uniformity of the Al_2_O_3_ coating.

### 3.2. Adhesive Strength of SCSSFFC/Al_2_O_3_

#### 3.2.1. Mechanical Shock Performance of SCSSFFC/Al_2_O_3_

**Figure 7:**
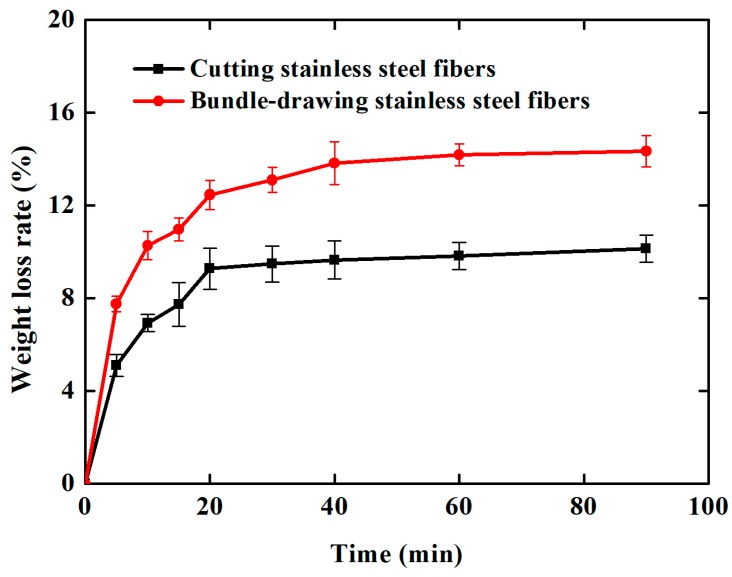
Weight loss rate-time curves of samples during ultrasonic vibration testing.

**Figure 8:**
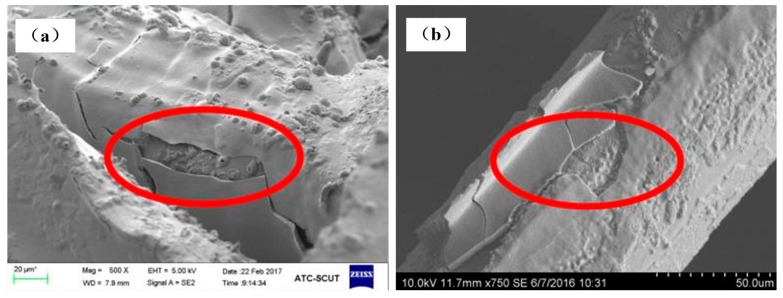
SEM of samples for 60 min ultrasonic adhesion test: (**a**) made of cutting fibers; (**b**) made of commercial drawing fibers.

The Al_2_O_3_ coating weight loss rate time curves are shown in Figure 7 for the ultrasonic vibration testing. Whether the samples are made of cutting or commercial bundle-drawing stainless steel fibers, the weight loss rates of the Al_2_O_3_ coating all increase linearly in the initial ten minutes of the ultrasonic vibration test. However, the slope of SCSSFFC/Al_2_O_3_ is significantly smaller than that of SBDSSFFC/Al_2_O_3_. Thereafter, the increase in the weight loss rate of Al_2_O_3_ coatings becomes gradually smaller. After testing for thirty minutes, the weight loss rate of SCSSFFC/Al_2_O_3_ approaches a plateau with a very small slope, while the weight loss rate of SBDSSFFC/Al_2_O_3_ reaches a plateau with the same slope until the testing has lasted for sixty minutes. At this time, the weight loss rate of SBDSSFFC/Al_2_O_3_ reaches 14.33% and the weight loss rate of SCSSFFC/Al_2_O_3_ is only 10.13%. Figure 8a,b presents the morphologies of samples made by cutting and bundle-drawing stainless steel fibers, respectively, after ultrasonic vibration testing for sixty minutes. Several crevices can be observed in Figure 8a but the coating has not yet peeled off; while in Figure 8b, it can be seen that many small crevices occur and the coating fragmentized and part of them has fallen off. The results indicate that the rough morphology of cutting stainless steel fibers is quite helpful in promoting the adhesive strength of the Al_2_O_3_ coating to the supports. 

#### 3.2.2. Thermal Shock Performance of SCSSFFC/Al_2_O_3_

**Figure 9:**
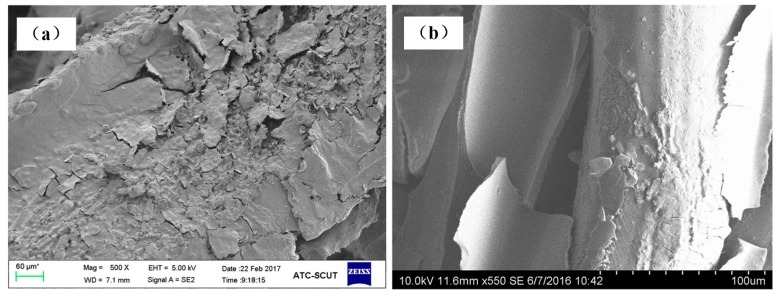
SEM of samples for the ninth thermal shock test: (**a**) made of cutting fibers; (**b**) made of commercial drawing fibers.

**Figure 10:**
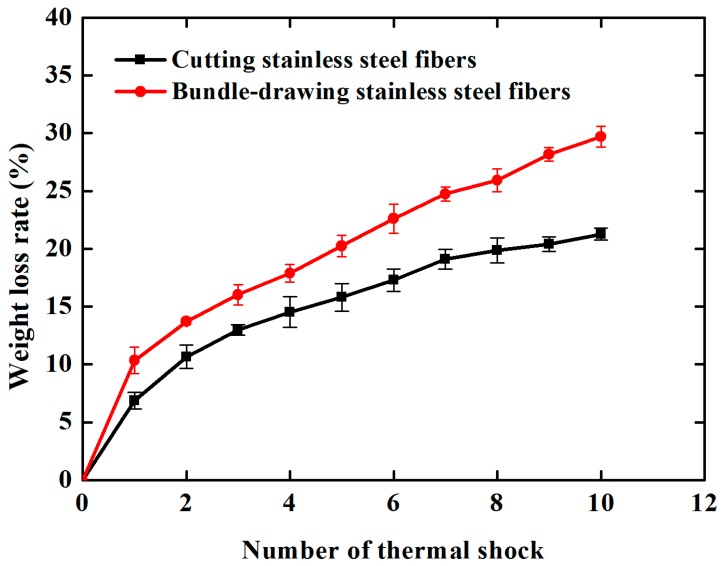
Weight loss rate-number of thermal shock curves of samples_._

Figure 9a,b shows the morphologies of samples made of cutting and bundle-drawing stainless steel fibers after the ninth thermal shock test. It appears from Figure 9a that many small pieces can be observed but the coating still attaches to the surface of SCSSFFC/Al_2_O_3_, while for SBDSSFFC/Al_2_O_3_, many large chunks occur and the coating has almost completely fallen off in bulk from Figure 9b. As shown in Figure 10, the Al_2_O_3_coating weight loss rate-number of thermal shock is presented for the thermal shock testing. Whether samples are made of cutting or bundle-drawing stainless steel fibers, the weight loss rates of the Al_2_O_3_ coating increase continuously for both as the number of thermal shocks increase. However, the slope of SCSSFFC/Al_2_O_3_ is substantially smaller than that of SBDSSFFC/Al_2_O_3_. After the tenth thermal shock, the weight loss rate of SBDSSFFC/Al_2_O_3_ is 29.70%, while the weight loss rate of SCSSFFC/Al_2_O_3_ is only 21.28%, which is a decrease of 8.42% compared with the SBDSSFFC/Al_2_O_3_. Therefore, the experimental results show that the rough morphology of the cutting fiber is strongly beneficial for improving the adhesive strength of Al_2_O_3_ coating to supports. 

### 3.3. Flow Characteristics of SCSSFFC/Al_2_O_3_

#### 3.3.1. Permeability

**Figure 11:**
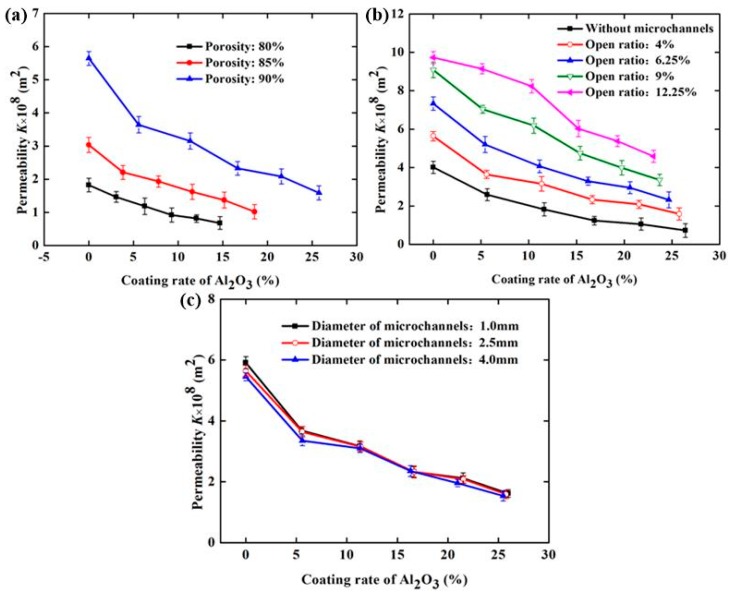
Permeability of SCSSFFC/Al_2_O_3_ with different Al_2_O_3_ coating rates under different structure parameters of SCSSFFC: (**a**) porosity (*α* = 4%, *d* = 2.5 mm); (**b**) open ratio (*ε* = 90%, *d* = 2.5 mm); (**c**) diameter of microchannel (*ε* = 90%, *α* = 4%).

Figure 11 demonstrates the effect of the Al_2_O_3_ coating rate and the structural parameters of SCSSFFC/Al_2_O_3_ on permeability when velocity of air is 0.3–1.5 m/s. From Figure 11, the Al_2_O_3_ coating rate has a significant influence on the permeability of SCSSFFC/Al_2_O_3_ and the permeability decreases with the increase in the Al_2_O_3_ coating rate. Compared with the SCSSFFC without an Al_2_O_3_ coating (*ε* = 90%, *α* = 4% and *d* = 2.5 mm), the permeability decreases by 35.46%, 44.1%, 58.68%, 63.1% and 71.8%, when the Al_2_O_3_ coating rate of SCSSFFC/Al_2_O_3_ is 5.60%, 11.36%, 16.66%, 21.56% and 25.76%, respectively.

As for the effect of the structural parameters, Figure 11a indicates that the permeability of SCSSFFC/Al_2_O_3_ is mainly affected by the porosity and open ratio but the internal microchannel diameter has little influence on permeability. For example, the permeability is 3.15 × 10^−8^, 6.16 × 10^−8^ and 8.2 × 10^−8^ m^2^ for the same 10% coating rate, when the open ratios of SCSSFFC/Al_2_O_3_ (*ε* = 90% and *d* = 2.5mm) are 4%, 6.25% and 12.250%, respectively. However, regardless of the internal microchannel diameter of SCSSFFC (1, 2.5 or 4 mm), the permeability of SCSSFFC/Al_2_O_3_ (*ε* = 90%, *α* = 4% and *d* = 2.5 mm) with a 25% coating rate is approximately 1.60 × 10^−8^ m^2^. 

To predict the permeability of SCSSFFC/Al_2_O_3_, a correction coefficient (*C_k_*) is suggested for the characterization of the effect of the coating rate and structural parameters on the permeability. The correlations for the permeability of SCSSFFC/Al_2_O_3_ based on the Dietrich correlations [32] is modified by the regression analysis of the experimental data and is expressed as follows:

(15) $$K=C_{K}\frac{\varepsilon \cdot d_{h}^{2}}{110}$$

(16) $$C_{K}=\left[(8.753\varepsilon -9.4028)\beta -1.982\varepsilon +2.5274\right]\cdot (5.1052\alpha +0.7886)$$

where *C_k_* is correction coefficient, *β* is coating rate of Al_2_O_3_, *ε* is porosity.

The average error between calculated values and experimental results of permeability is 5.3916%. Therefore, the permeability for the SCSSFFC/Al_2_O_3_ based on the Dietrich correlation agrees well with the experimental results. 

#### 3.3.2. Inertial Coefficient

**Figure 12:**
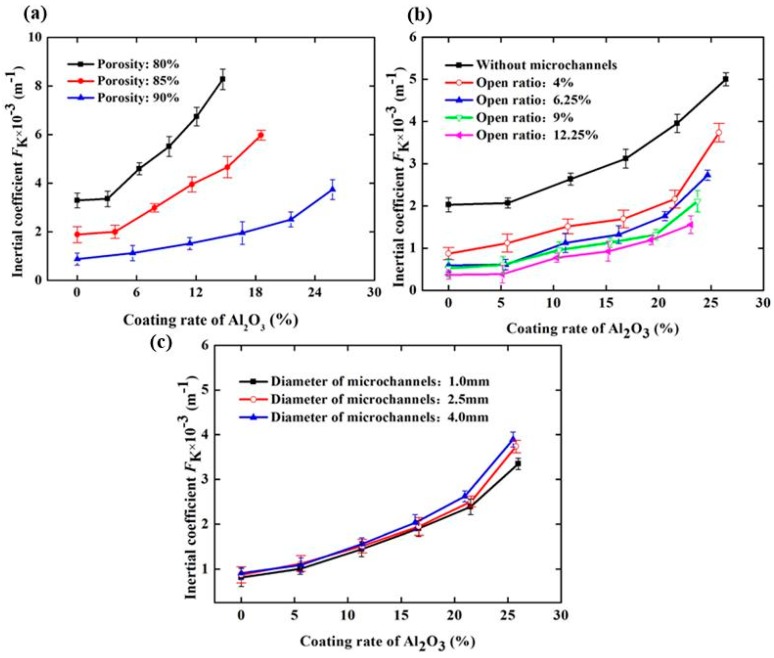
Inertial coefficient of SCSSFFC/Al_2_O_3_ with different Al_2_O_3_ coating rates under different structural parameters of SCSSFFC: (**a**) porosity (*α* = 4%, *d* = 2.5 mm); (**b**) open ratio (*ε* = 90%, *d* = 2.5 mm); (**c**) diameter of microchannel (*ε* = 90%, *α* = 4%).

To better describe the change in the flow characteristics of SCSSFFC/Al_2_O_3_ caused by the Al_2_O_3_ coating rate and structural parameters, the effect of the Al_2_O_3_ coating rate and structural parameters on the inertial coefficient of SCSSFFC/Al_2_O_3_ is investigated, as shown in Figure 12. In Figure 12, it can be seen that the inertial coefficient significantly increases with the coating rate because of the improved drag effect and tortuosity of the flow channels after coating. Compared with the SCSSFFC without an Al_2_O_3_ coating (*ε* = 80%, *α* = 4% and *d* = 2.5 mm), the inertial coefficient of SCSSFFC/Al_2_O_3_ increases by 0.471, 0.7834, 1.165 and 1.59 times, when the coating rate is 6.25%, 9.16%, 12.3% and 14.8% respectively. Furthermore, the inertial coefficients of SCSSFFC/Al_2_O_3_ mainly depend on the open ratio and the porosity, as shown in Figure 12. For the effect of the internal microchannel diameter, the results indicate that the diameter has little effect on the inertial coefficient. For example, the inertial coefficient of the SCSSFFC/Al_2_O_3_ (*ε* = 90%, *α* = 4% and *d* = 2.5 mm) with a 25% coating rate is approximately 3.74 × 10^3^ m^−1^, whether the honeycomb channel diameter of the SCSSFFC is 1 mm, 2.5 mm or 4 mm.

Another correction coefficient (*C_F_*) is proposed to characterize the effect of the coating rate and structural parameters on the inertial coefficient for assessing the inertial coefficient. The modified correlation is given based on the Dietrich correlation as follows:

(17) $$F_{K}=C_{F}\frac{1.45}{\varepsilon^{2}\cdot d_{h}}$$

(18) $$\begin{array}{ll} C_{F}= & \left[(-18.454\varepsilon +24.574)\beta -4.167\varepsilon +4.3245\right]\cdot (-2.2757\alpha +1.0874)\\ & \cdot (79.136\alpha^{2}-16.789\alpha +1.5117) \end{array}$$

where *C_F_* is correction coefficient for SCSSFFC/Al_2_O_3_. 

The average error between calculated values and experimental results of inertial coefficient is 6.4766%. Therefore, the modified equation is valid to predict the inertial coefficient for rough SCSSFFC/Al_2_O_3_. 

## 4. Discussion

### 4.1. Comparison with Smooth SBDSSFFC/Al_2_O_3_

**Figure 13:**
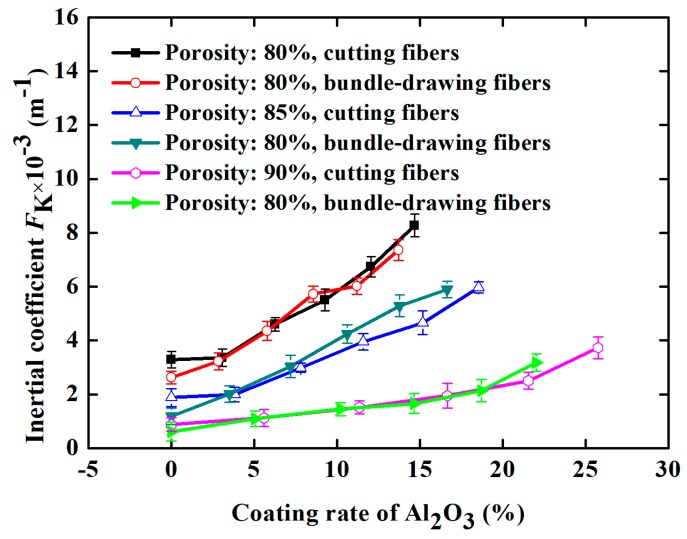
Inertial coefficient of the samples with different Al_2_O_3_ coating.

**Figure 14:**
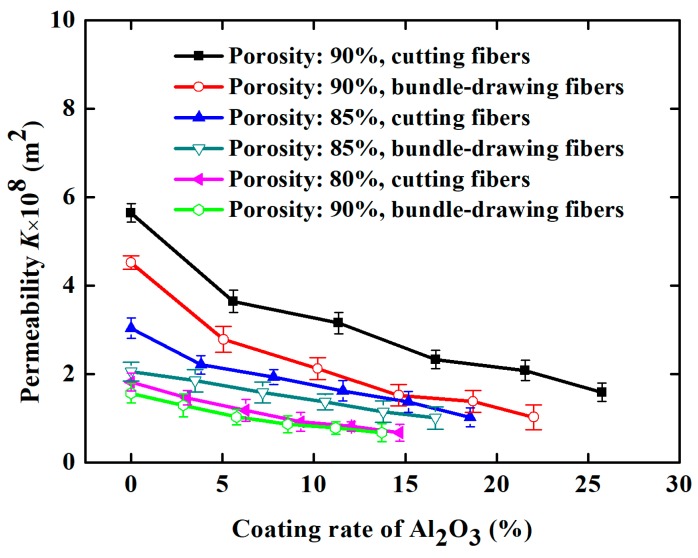
Permeability of samples with different Al_2_O_3_ coating rates.

For comparison, SBDSSFFC/Al_2_O_3_ samples (*ε* = 90%, *α* = 4% and *d* = 2.5 mm) made of bundle-drawing fibers available commercially are also prepared and tested using the same preparation processes. Figure 13 indicates that the inertial coefficients of both kinds of samples are nearly the same, regardless of whether the samples are made of the cutting stainless steel fibers or commercial bundle-drawing fibers. However, SCSSFFC/Al_2_O_3_ exhibits a large permeability compared with that of SBDSSFFC/Al_2_O_3_, as shown in Figure 14. For example, data for the permeability of SCSSFFC/Al_2_O_3_ is approximately 57.14% higher than that of SBDSSFFC/Al_2_O_3_ for the same 10% coating rate and 90% porosity. Therefore, the rough cutting stainless steel fiber significantly improves the permeability of SCSSFFC/Al_2_O_3_ compared with that of SBDSSFFC/Al_2_O_3_.

### 4.2. Comparison with Previous Work

Because of their excellent comprehensive performance, ceramic and metallic foam materials are also selected for comparison. The permeability and inertial coefficients of SCSSFFC/Al_2_O_3_ and the foam materials reported in previous work [32,33] are listed in Table 1, in which the comparative data are calculated from the correlations of the foam materials. Table 1 indicates that the data for SCSSFFC/Al_2_O_3_ show an approximately 3.1–67.34% rise in permeability compared with the foam materials with the same porosity. Moreover, the inertial coefficients of SCSSFFC/Al_2_O_3_ are approximately 0.45–5.75 times higher than those of the compared foam materials for the same porosity. Therefore, SCSSFFC/Al_2_O_3_ has both comparable permeability and excellent inertial coefficients which can serve as a good choice for catalyst supports.

## 5. Conclusions

(1) A novel sintered cutting stainless steel fiber felt with internal channels (SCSSFFC) made by cutting fiber is developed. SCSSFFC has a jagged and rough surface microstructure and possesses a high specific surface area approximately 2.4 times larger than that of SBDSSFFC. 

(2) Based on ultrasonic vibration and thermal shock tests, the rough morphology of the cutting fibers strongly improves the adhesive strength of the Al_2_O_3_ coating to supports. Compared with SBDSSFFC/Al_2_O_3_, the weight loss rates of SCSSFFC/Al_2_O_3_ decrease by 4.2% and 8.42%. 

(3) The rough cutting stainless steel fiber significantly improves the permeability of SCSSFFC/Al_2_O_3_ compared with that of the commercial bundle-drawing fibers. Data for the permeability of SCSSFFC/Al_2_O_3_ are approximately 57.14% higher than those of SBDSSFFC/Al_2_O_3_. 

(4) SCSSFFC/Al_2_O has both comparable permeability and excellent inertial coefficients. Compared with the reported foam materials, data for SCSSFFC/Al_2_O_3_ show an approximately 3.1–67.34% rise in permeability. Moreover, the inertial coefficients of SCSSFFC/Al_2_O are approximately 0.45–5.75 times higher than those of the compared foam materials. 

## Figures and Tables

**Table 1:** Comparison of permeability and inertial coefficients of the SCSSFFC/Al_2_O_3_ and foam materials.

ε	Current Experimental Data				Topin et al. [32]		Dietrich [33]	
	α	β	K × 10^8^ m^2^	F_k_ × 10^−3^ m^−1^	K × 10^8^ m^2^	F_k_ × 10^−3^ m^−1^	K × 10^8^ m^2^	F_k_ × 10^−3^ m^−1^
0.80	4%	2.5%	1.5	3.3	1.1	1.318	1.1	0.618
0.85	4%	0%	3.0	2.0	2.91	0.742	2.91	0.348
0.90	12.25%	10%	8.2	0.76	4.9	0.523	4.9	0.24

## Nomenclature

*K*	permeability (m^2^)
*F*_K_	inertial coefficient (m^−1^)
*M*	mass of sample (kg)
Δ*p*	pressure drop (Pa)
*D*	sample diameter (m)
*d*	channel diameter (m)
*H*	thickness of sample (m)
*N*	channel number
*u*	air velocity (m/s)
*C_k_*	correction coefficient of permeability
*C_F_*	correction coefficient of inertial coefficient
Greek symbol	
*ε*	porosity, dimensionless
*ρ*	density of air (kg/m^3^)
*ρ*′	density of 1Cr18Ni9Ti (kg/m^3^)
*μ*	dynamic viscosity of air (Pa s)
*α*	open ratio, dimensionless, dimensionless
*β*	coating rate of sample, dimensionless
*φ*	weight loss rate of sample, dimensionless
